# Are Dietary Cholesterol Intake and Serum Cholesterol Levels Related to Nonalcoholic Fatty Liver Disease in Obese Children?

**DOI:** 10.1155/2012/572820

**Published:** 2012-07-03

**Authors:** Dimitrios Papandreou, Zaharoula Karabouta, Israel Rousso

**Affiliations:** ^1^2nd Department of Pediatrics, School of Medicine and Ahepa General Hospital, Aristotle University of Thessaloniki, Street Kiriakidi 1, 54636 Thessaloniki, Greece; ^2^Department of Life and Health Sciences, University of Nicosia, Makedonitissas Avenue, 1700 Nicosia, Cyprus

## Abstract

*Background*. Nonalcoholic fatty liver disease (NAFLD) in children has been recognized as a major health burden. Serum lipids as well as dietary cholesterol (DC) intake may positively relate to development of NAFLD. The purpose of this study was to investigate anthropometric, biochemical, and dietary intake parameters of obese Greek children with and without NAFLD. *Materials and Methods*. Eighty-five obese children aged 8–15 (45 boys/40 girls) participated in the study. NAFLD was diagnosed by ultrasonography (US) in all subjects. Liver indexes were measured in all children. A 3-day dietary was recorded for all subjects. *Results*. 38 out of 85 children (44.7%) were found to have fatty liver. Obese children with increased levels of TC (95% CI: 1.721–3.191), low density lipoprotein (LDL) (95% CI: 1.829–3.058), and increased dietary cholesterol intakes (95% CI: 1.511–2.719) were 2.541, 2.612, and 2.041 times more likely to develop NAFLD compared with the children without NAFLD. *Conclusion*. The present study showed that TC, LDL, and DC were the strongest risk factors of development of NAFLD. Reducing body weight and dietary cholesterol intakes as well as decreasing serum TC and LDL levels are urgently necessary in order to prevent NAFLD and possible other health implications later in life.

## 1. Introduction

Nonalcoholic fatty liver disease (NAFLD), which encompasses a broad spectrum of liver disorders ranging from simple hepatic steatosis to steatohepatitis (NASH) and cirrhosis, is currently the most common cause of chronic liver disease and abnormal liver function tests in Western countries [1]. The development of hepatic steatosis is considered to be associated with an excess intake of calories, visceral obesity, and insulin resistance, which result in an increased release of free fatty acids from adipocytes and increased rates of fatty acid synthesis in the liver [1]. Therefore, nutritional management and therapeutic exercise are fundamental steps to treat NAFLD.

The “two-hit theory” is increasingly being adopted to explain the pathogenesis of NAFLD and NASH [2]. In this theory, the first hit consists of the accumulation of fatty acids/triglycerides in the liver, while the second hit involves oxidative stress, mitochondrial dysfunction, and inflammation, which ultimately cause liver damage.

Hepatic lipid homeostasis represents a balance between lipid uptake, synthesis, catabolism, and secretion. Therefore, steatosis, a typical characteristic of NAFLD, is expected to be caused by disordered lipid metabolism, particularly inhibition of fatty acid oxidation and enhanced lipogenesis. Many factors involved in hepatic lipid metabolism pathways have been identified, even though the precise cellular networks are not fully elucidated. In humans with NAFLD, cholesterol uptake in the form of LDL is limited by the intracellular accumulation of fatty acid and cholesterol, while fatty acid and cholesterol synthesis are upregulated in the NAFLD liver suggesting that the feedback system regulating and clearing up intracellular lipids levels is disrupted in NAFLD [3].

The prevalence of NAFLD in children remains unknown because of the lack of population-based studies and reliable screening tools. The available data suggests a prevalence range from 2.6% to 9.6% among children and adolescents in the United States and Asia which may reach to 12–80% in overweight and obese children [4]. Even though there is no consensus for the treatment for NAFLD in children, a recent review shoed that many studies have been attempted to treat NAFLD using weight loss and exercise, antioxidants, and pharmacological agents [5].

Some studies have been suggesting that high-fat, high-fat plus low-protein, high-carbohydrate, and/or high-cholesterol diets are the main causes of NAFLD [6–8]. The reason for this is that cholesterol overload can upregulate gene expression and activate fatty acid synthesis by increasing oxysterol levels, metabolites of cholesterol that act as agonists for these genes indicating that excess cholesterol intake (i.e., cholesterol supply) itself can be a strong stimulant for the development of steatosis [9].

The purpose of this study was to investigate possible differences of certain parameters such as anthropometric, biochemical, and dietary of obese Greek children with and without NAFLD.

## 2. Materials and Methods

Eighty-five (85) obese children aged 8–15 y (45 boys/40 girls) were initially referred for obesity assessment to the outpatient 2nd Pediatric Department of Aristotle University of Thessaloniki, between January 2007 and January 2011. All children were screened for parameters of NAFLD. Written, informed consent was also obtained from each child's parents who participated in the study.

### 2.1. Anthropometrics and Biochemical Indexes

Anthropometrical indices such as height, weight, Tanner stage, and waist circumference were assessed in all children. Obesity was defined by International Obesity Task Force criteria for age and sex [10]. BMI was calculated by dividing weight (kg) by height squared (m^2^). Biochemical indices such as liver transaminases (ALT, AST), lipid profile, total cholesterol (TC), low density lipoprotein (LDL), high density lipoprotein (HDL), and triglycerides (TG) were measured in all children. All children had normal serum levels of alanine aminotransferase (ALT) <40 U/L and aspartate aminotransferase (AST) <37 U/L. Participants were free of a history of clinical evidence of diabetes, cardiovascular, liver disease, hepatits B or C, use of alcohol, drugs, or other disease (e.g., Wilson's disease, autoimmune hepatitis). NAFLD was diagnosed by ultrasonography (US) and was classified as having no or moderate hepatic steatosis (Grade II). The examination was performed by a radiologist with a convex probe (3.5–5 MHZ) with Elegra-Siemens equipment. The echogenicity of the liver was estimated in a longitudinal US slice, showing the liver parenchyma and the neighboring right kidney [11]. 

Insulin resistance (IR) was calculated by means of the homeostasis model assessment (HOMA). IR was defined by HOMA >3.2 *μ*U/mL [12].

### 2.2. Dietary Intake

A validated dietary intake [13] was used to collect information for a total of 3 days (2 weekdays and 1 weekend day). Study participants together with their parents were asked to describe the type and amount of food consumed after a detailed explanation and guidance provided by a registered dietician of our clinic. To improve the accuracy of food description, food models were used to describe the portion sizes. The dietary record was then analyzed using a software program (Sciencetech Diet 200A, Science Technologies, Athens, Greece, 2001) which consisted of more than 2500 Greek food items and recipes. 

### 2.3. Statistical Analysis

Standard descriptive statistics were used for all patient characteristics. Data for continuous variables is expressed as mean values ± standard deviation. *t*-test was used to compare characteristics of obese children with and without NAFLD. Partial correlation was estimated by using Spearman's correlation coefficient. Logistic regression analysis was used to estimate the relationship between ultrasound findings (normal or pathological) with HOMA, BMI, WC, HDL, LDL, dietary cholesterol, saturated fatty acid, sugar, and fiber consumed daily. *P* values < 0.05 were considered statistically significant, while all confidence intervals (C.I.) represent 95% intervals.

## 3. Results

Based on ultrasonography, 38 out of 85 children (44.7%) were found to have fatty liver. 

Anthropometric and biochemical characteristics of all subjects were presented in Table 1. Children with NAFLD were having higher BMI, WC TC, and LDL and lower HDL levels compared to children without NAFLD. In addition IR was significantly higher in obese children with NAFLD.

The dietary intakes of energy and major nutrients in the three categories are reported in Table 2. SFA, cholesterol, and sugar intakes were significantly higher in group with NAFLD compared with the group without NAFLD.

In Spearman's correlation model, BMI, WC, TC, LDL, and dietary cholesterol were found to be positively correlated with NAFLD, while HDL levels were negatively associated with NAFLD (Table 3).

In logistic regression analysis, obese children with increased levels of TC (95% CI: 1.721–3.191), low density lipoprotein (LDL) (95% CI: 1.829–3.058), and increased dietary cholesterol intakes (95% CI: 1.511–2.719) were 2.541, 2.612, and 2.041 times more likely to develop NAFLD compared with the obese children without NAFLD (Table 4).

## 4. Discussion

In the current study, we presented clinical data of 85 obese children with and without NAFLD proven by ultrasonography and its association with anthropometric and clinical parameters as well as with dietary intakes. The prevalence of our sample data was 38/85 (44.7%) which is between the prevalence (12–80%) found by other authors in a review study of different regions of the world such as Europe, America, and Asia [4]. 

It has been reported that the degree of steatosis in NAFLD is proportional to the degree of obesity [14]. BMI and WC were found in our study positively correlated with hepatic fat content. However, none of them were of the strongest risk factors of developing NAFLD in the logistic regression model analysis, suggesting that obesity and splanchnic fat distribution might also be effects of insulin resistance, rather than being directly involved in the etiology of fatty liver.

In our study the obese children with NAFLD were presented to have increased levels of insulin resistance, dyslipidemia, and WC. NAFLD is strongly associated with obesity, insulin resistance, hypertension, and dyslipidemia and is now regarded as the liver manifestation of the metabolic syndrome (MetS) [15], a highly atherogenic condition.

Diets rich in fatty acids mainly saturated and trans-fatty acids, as well as carbohydrate-rich diets, favor an acute increase in IR independent of adiposity [16]. High SFA intake may also promote steatohepatitis directly by modulating hepatic triglyceride accumulation and oxidative activity as well as indirectly by affecting insulin sensitivity and postprandial triglyceride metabolism [17]. SFA consumption where found to be significantly higher in children with NAFLD compared with the children without NAFLD.

The consumption of sugar has been linked to risks for obesity, diabetes, metabolic syndrome, fatty liver, and heart disease, possibly by providing excess calories and large amounts of rapidly absorbable sugars [18, 19]. Our results showed that obese children with NAFLD had significantly higher intakes compared to children without NAFLD.

The most important finding of our study was that the children with NAFLD consumed significantly higher levels of dietary cholesterol compared to children without NAFLD. In addition, the total serum cholesterol levels of the same group were also found to significantly higher, compared with the group without NAFLD.

Observational studies have been conflicting with regard to our findings. Some studies did not demonstrate different dietary intakes of cholesterol between NAFLD patients and controls [20, 21]. However, Musso et al. [17] did demonstrate a higher cholesterol consumption among normal weight NASH patients versus BMI matched controls. A recent study supported the role of dietary cholesterol in NAFLD. In this study, 12 normal weight NAFLD patients were compared to 44 obese NAFLD patients. A characteristic feature was that dietary cholesterol intake was significantly higher, while the intake of polyunsaturated fatty acids (PUFAs) was significantly lower, in the nonobese group. Similar differences were noted in comparison to 15 healthy nonobese controls. Therefore, this altered cholesterol and PUFA intake may be associated with the development of NAFLD in nonobese patients [9]. In addition, studies using nonobese animal models have confirmed that a diet high in cholesterol can induce NASH [22].

Obese children with NAFLD should be informed that a healthy diet has benefits beyond weight reduction. They should be advised to reduce saturated/trans-fat and increase polyunsaturated fat with special emphasize on omega-3 fatty acids. They should reduce added sugar to its minimum, and increase fiber intake. Physical activity should be integrated into behavioral therapy in NAFLD, as even small gains in PA and fitness may have significant health benefits. A combination of educational, behavioral, and motivational strategies is required to help patients achieve lifestyle change.

## 5. Conclusion 

The present study showed that TC, LDL, and DC were the strongest risk factors of development of NAFLD. Reducing body weight and dietary cholesterol intakes as well as decreasing serum TC and LDL levels is urgently necessary in order to prevent NAFLD and possible other health implications later in life.

## 6. Limitations

Limitations of our study include the small size of the participants and the lack of liver biopsy. We also did not present activity level of patients as well as socioeconomic data.

## Figures and Tables

**Table 1:** Clinical characteristics of subjects based on fatty liver.

	Absent (*n* = 47)	Moderate (Grade II) (*n* = 38)	*P* value
Sex (M/F)	25/22	20/18	0.451
Age (y)	11 ± 2.1	11.5 ± 1.1	0.369
BMI (kg/m^2^)	25 ± 3.6	31 ± 2.9	**0.001**
WC (cm)	85.2 ± 11.2	97 ± 10.9	**0.001**
ALT (U/L)	22.7 ± 6.2	26 ± 4.1	0.942
AST (U/L)	21.1 ± 7.4	25.1 ± 3.2	0.212
TC (mg/dL)	168.7 ± 27.4	179 ± 19.8	**0.006**
HDL (mg/dL)	50.3 ± 13.1	40 ± 12.1	**0.002**
LDL (mg/dL)	103.7 ± 22.1	94 ± 9.6	**0.043**
TG (mg/dL)	110.2 ± 54.3	109.1 ± 60	0.294
CRP	2.2 ± 2.8	2.4 ± 2.6	0.591
HOMA-IR	4.3 ± 1.9	6.2 ± 2.1	**0.001**

Values are presented as means ± SD. Statistical significant difference (*P*  < 0.05). Abbreviations: BMI: body mass index, WC: waist circumference, ALT: alanine aminotransferase, AST: aspartate aminotransferase, TC: total cholesterol, LDL: low density lipoprotein, HDL: high density lipoprotein, TG: triglycerides, CRP: C-reactive protein, HOMA-IR: homeostasis model assessment of insulin resistance.

**Table 2:** Dietary intake of subjects based on fatty liver.

	Absent (*n* = 47)	Moderate (Grade II) (*n* = 38)	*P* value
Energy (kcal)	2444 ± 539	2605 ± 489	0.641
PRO (g)	103.1 ± 27.7	97 ± 23.3	0.323
CHO (g)	244.5 ± 67.5	276.1 ± 51.9	0.051
FAT (g)	139.7 ± 45.1	138 ± 41.9	0.079
SFA (g)	45.1 ± 12.6	61.9 ± 11.8	**0.032**
CHOL (mg)	321 ± 29.7	378 ± 28.7	**0.001**
Sugar (g)	10.8 ± 15.3	21 ± 12.9	**0.002**
Fiber (g)	20.0 ± 8.0	15 ± 9.1	0.081

Values are presented as means ± SD. Statistical significant difference (*P*  < 0.05). Abbreviations: PRO: protein, CHO: carbohydrates, SFA: saturated fatty acid, CHOL: dietary cholesterol.

**Table 3:** Parameters associating with NAFLD by Spearman's correlation coefficient.

Parameters	*r*	*P* value
BMI (kg/m^2^)	0.521	**0.001**
WC (cm)	0.296	**0.001**
HOMA-IR	0.175	0.501
TC (mg/dL)	0.533	**0.001**
HDL (mg/dL)	−0.281	**0.031**
LDL (mg/dL)	0.419	**0.021**
SFA (g)	0.081	0.092
CHOL (mg)	0.312	**0.009**
Sugar (g)	0.109	0.290

Statistical significant difference (*P*  < 0.05). Abbreviations: BMI: body mass index, WC: waist circumference, HDL: high density lipoprotein, HOMA-IR, homeostasis model assessment of insulin resistance, TC: total cholesterol, SFA: saturated fatty acids, CHOL: dietary cholesterol.

**Table 4:** Parameters associating with NAFLD by logistic regression analysis^∗^.

Parameters	Odds Ratio	95% CI	*P* value
BMI (kg/m^2^)	1.141	(0.871–1.645)	0.349
WC (cm)	1.192	(0.941–1.779)	0.452
HOMA-IR	1.488	(1.108–1.877)	0.229
TC (mg/dL)	**2.541**	(1.721–3.191)	**0.001**
HDL (mg/dL)	1.228	(0.877–1.524)	0.612
LDL (mg/dL)	**2.612**	(1.829–3.058)	**0.001**
SFA (g)	1.211	(0.544–1.612)	0.053
CHOL (mg)	**2.041**	(1.511–2.719)	**0.009**
Sugar (g)	0.448	(0.156–0.997)	0.319

Statistical significant difference (*P*  < 0.05). ^∗^Adjusted for age, gender, and energy intake. Abbreviations: BMI: body mass index, WC: waist circumference, HDL: high density lipoprotein, HOMA-IR: homeostasis model assessment of insulin resistance, CHOL: dietary cholesterol, SFA: saturated fatty acids.
